# Retraction: Tempol Treatment Reduces Anxiety-Like Behaviors Induced by Multiple Anxiogenic Drugs in Rats

**DOI:** 10.1371/journal.pone.0292639

**Published:** 2023-10-04

**Authors:** The PLOS ONE Editors

Following the publication of this article [1], concerns were raised regarding the results in multiple figures. Specifically,

- The following panels appear similar despite representing different conditions:
  - β-actin panels in Figs 7D and 7K.
  - β-actin panels in Figs 8E and 9D.
  - c-fos panels in Figs 9D and 9E.
- Data tables are not consistent with the reported number of samples in multiple graphs.

The corresponding author provided original uncropped western blots underlying some of the results in Figs 7–9. In relation to the three pairs of similar panels representing different conditions in Figs 7D and 7K, 8E and 9D, and 9D and 9E, the corresponding author stated that the pairs are not the same. Their responses and the original uncropped images did not resolve the concerns regarding similarity between these blots.

The corresponding author provided data tables underlying all graphs other than those in Fig 5 and Fig 6D. Data tables underlying the graphs in Figs 3A, 6C, 7, 8 and 9 contain less samples than indicated in the corresponding figure legends. Data tables underlying the graphs in Fig 4 contain inconsistent numbers of samples for the Tempol and FG-7142 groups. Additionally, many data tables contain values labelled as outliers that were excluded from analyses. The corresponding author’s responses did not resolve these concerns which question the accuracy of data reporting and the reliability of the results.

In light of the concerns affecting multiple figure panels that question the reliability of these data, the *PLOS ONE* Editors retract this article.

SS did not agree with the retraction and stands by the article’s findings. All other authors either did not respond directly or could not be reached.
